# Basic research on postoperative cognitive dysfunction in the past decade: a bibliometric analysis

**DOI:** 10.3389/fnagi.2025.1529860

**Published:** 2025-03-19

**Authors:** Hongwei Wu, Jiannan Song, Zhanfei Hu, Haibo Li, Qi Zhou, Congcong Dai, Yi Gao, WanLi Ma

**Affiliations:** ^1^Chifeng Clinical Medical College of Inner Mongolia Medical University, Chifeng, China; ^2^Department of Anesthesiology, Chifeng Municipal Hospital, Chifeng, China; ^3^Department of Anesthesiology, Beijing Shijitan Hospital, Capital Medical University, Beijing, China

**Keywords:** basic research, bibliometrics, postoperative cognitive dysfunction, CiteSpace, VOSviewer

## Abstract

**Objective:**

Postoperative cognitive dysfunction (POCD) is a prevalent complication following anesthesia and surgery that particularly affects elderly patients, and poses significant health risks. In recent years, there has been an increase in basic research on POCD, with a particular focus on its molecular mechanisms, which have become a prominent area of inquiry. However, no bibliometric analysis has been conducted in this field. This study aims to employ bibliometric methods to comprehensively summarize the current status and developmental trends of basic research on POCD, providing new ideas and strategies for future scientific investigations.

**Methods:**

Relevant literature published between January 1, 2014, and October 30, 2024, was retrieved from the Web of Science Core Collection. Eligible articles were exported in plain text format. The annual output of published papers and data on authors, countries/institutions, journals, keywords, co-cited journals, and co-cited literature were analyzed and visualized using Microsoft Excel, VOSviewer, and CiteSpace software.

**Results:**

A total of 479 papers from 13 countries were included, with a noticeable upward trend in publications over the past decade, particularly in the last 3 years. A total of 105 core authors published four or more papers, with Professor Zuozhiyi identified as the leading contributor. “The Journal of Neuroinflammation” emerged as the most prolific publication source, while Chinese scholars accounted for the highest number of contributions and Dutch scholars led in citations per article. The University of Virginia was the leading institution for publications. Analysis of research hotspots revealed “neuroinflammation,” “surgery,” “impairment,” “memory,” and “information” as frequently occurring keywords. Notably, “pyroptosis” was identified as a current research hotspot and “synaptic plasticity” as a rapidly emerging term. The top five cited journals were all ranked as Q1 journals, with “Anesthesiology” being the most cited. Within co-cited articles, the “hippocampal CA1 region” represented the largest cluster, and literature on “neuroinflammation” was a key reference in current discussions.

**Conclusion:**

Over the past decade, basic research on POCD has steadily increased, particularly among Chinese scholars. Bibliometric analysis revealed that the molecular mechanisms underlying POCD are likely crucial focuses of current and future research. This field holds significant potential for further development.

## 1 Introduction

Postoperative cognitive dysfunction (POCD) is a common complication following anesthesia and surgery is characterized by declines in memory, orientation, language expression, and social skills. Its prevalence can reach as high as 36.3% among younger individuals and 42.4% among elderly (Monk et al., 2008; Needham et al., 2017). In 2018, perioperative neurocognitive disorders (PNDs) were formally proposed as a comprehensive diagnostic category encompassing cognitive impairment or alterations detected either preoperatively or postoperatively. As a specific subtype of PND, POCD is defined by measurable cognitive deficits emerging between 30 days and 12 months following surgical procedures (Evered et al., 2018). This condition poses significant risks, with studies linking it to prolonged hospital stays, adverse prognoses, diminished postoperative quality of life, and increased mortality rates (Rundshagen, 2014). Currently, the mechanisms underlying POCD remain unclear; however, previous research has suggested potential associations with neuroinflammation, neuronal apoptosis, synaptic plasticity impairment, pain, and mitochondrial metabolic disorders (Gao et al., 2021; Ding et al., 2021; Yang et al., 2022; Yuan et al., 2022; Bowman et al., 2018). Furthermore, Campbell and Figueiro (2024) reviewed evidence indicating that postoperative circadian rhythm disorder (CRD) may induce POCD by disrupting sleep architecture. Wei et al. (2024) further demonstrated that preoperative gut microbiota dysbiosis may serve as a significant contributing factor to the development of POCD.

As one of the most widely utilized statistical methods today, bibliometric analysis evaluates academic productivity, delineates academic frontiers and hotspots, and predicts scientific trends within research domains by leveraging scientific literature databases and bibliometric attributes (Ninkov et al., 2022). VOSviewer and CiteSpace are among the most frequently employed bibliometric tools for literature data analysis and visualization, as they effectively assess structured content and the evolution of topics to provide a clearer understanding for readers (van Eck and Waltman, 2010; Chen, 2006). In recent years, interest in research on POCD has been steadily increasing; however, comprehensive bibliometric analyzes, particularly those focused on basic research in this area, remain sparse. Therefore, this paper aims to employ bibliometric methods to summarize existing basic research on POCD, explore its underlying molecular mechanisms, thoroughly analyze the research results and quantitative data in this field, and provide a visual map. The goal is to offer valuable insights for scholars interested in this research domain, thereby ultimately contributing to more effective prevention and intervention strategies for POCD in clinical practice.

## 2 Methods

### 2.1 Data extraction

The Web of Science is widely recognized as a high-quality database, that is valued by researchers for its extensive subject coverage, rigorous journal screening, and rich collection of literature resources. It is considered the most suitable database for bibliometric analysis (Ding and Yang, 2020; Thelwall, 2008). This paper utilized the Web of Science Core Collection (WoSCC) as the source for literature data. To ensure the comprehensiveness and accuracy of the retrieval results, the citation index was set to the Science Citation Index Expanded. The retrieval formula employed was TS = (“postoperative cognitive dysfunction”), with the time frame specified from January 1, 2014, to October 30, 2024. For the literature type, only articles are selected.

### 2.2 Data cleaning

**Figure 1:**
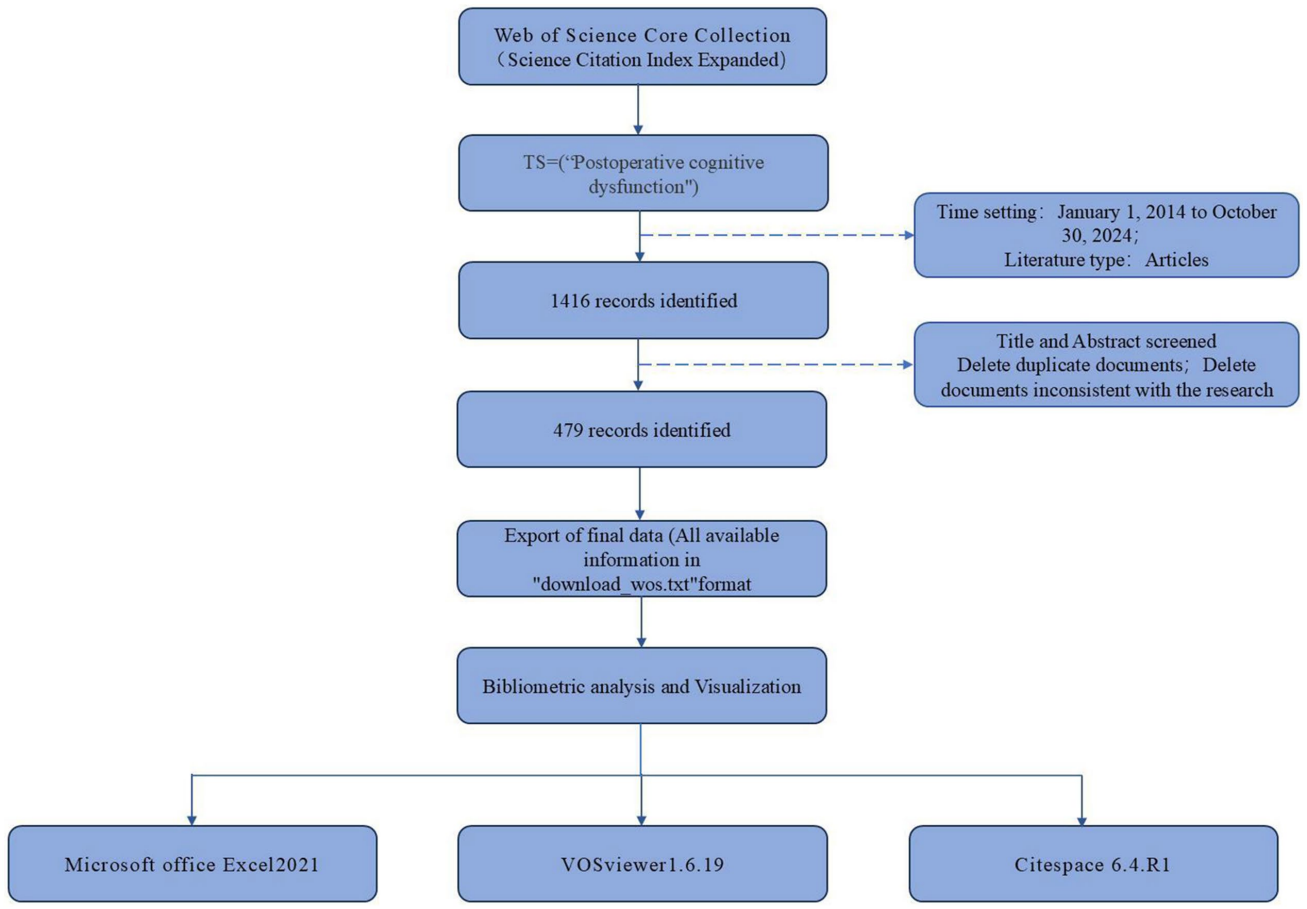
Flowchart of articles extraction and screening process.

A total of 1,416 articles were retrieved from the database, after which duplicate entries were removed. Titles and abstracts were reviewed, and only those works directly related to basic research POCD were selected, while studies focused on clinical research or meta-analyzes were excluded. This process resulted in the removal of 934 articles, leaving a total of 479 relevant articles for inclusion. These files were then exported in “plain text” format and saved as “download_wos.txt.” To ensure the reliability of the results, the data extraction and screening process was conducted by two independent researchers. For a detailed overview of this process, please refer to Figure 1.

### 2.3 Data processing

Microsoft Office Excel 2021, VOSviewer1.6.19, and CiteSpace 6.4.R1 were used to visually analyze the final exported literature; summarize the trends in the number of published papers, high-yield authors, countries, institutions, and journals; and analyze the co-occurrence, clustering of keywords and co-citation information.

## 3 Results

### 3.1 Trends of published documents

The 479 articles included in this study were written by 2,271 authors from 13 countries, 393 institutions or organizations, published in 182 journals, and cited 13,361 references from 10,609 authors.

**Figure 2:**
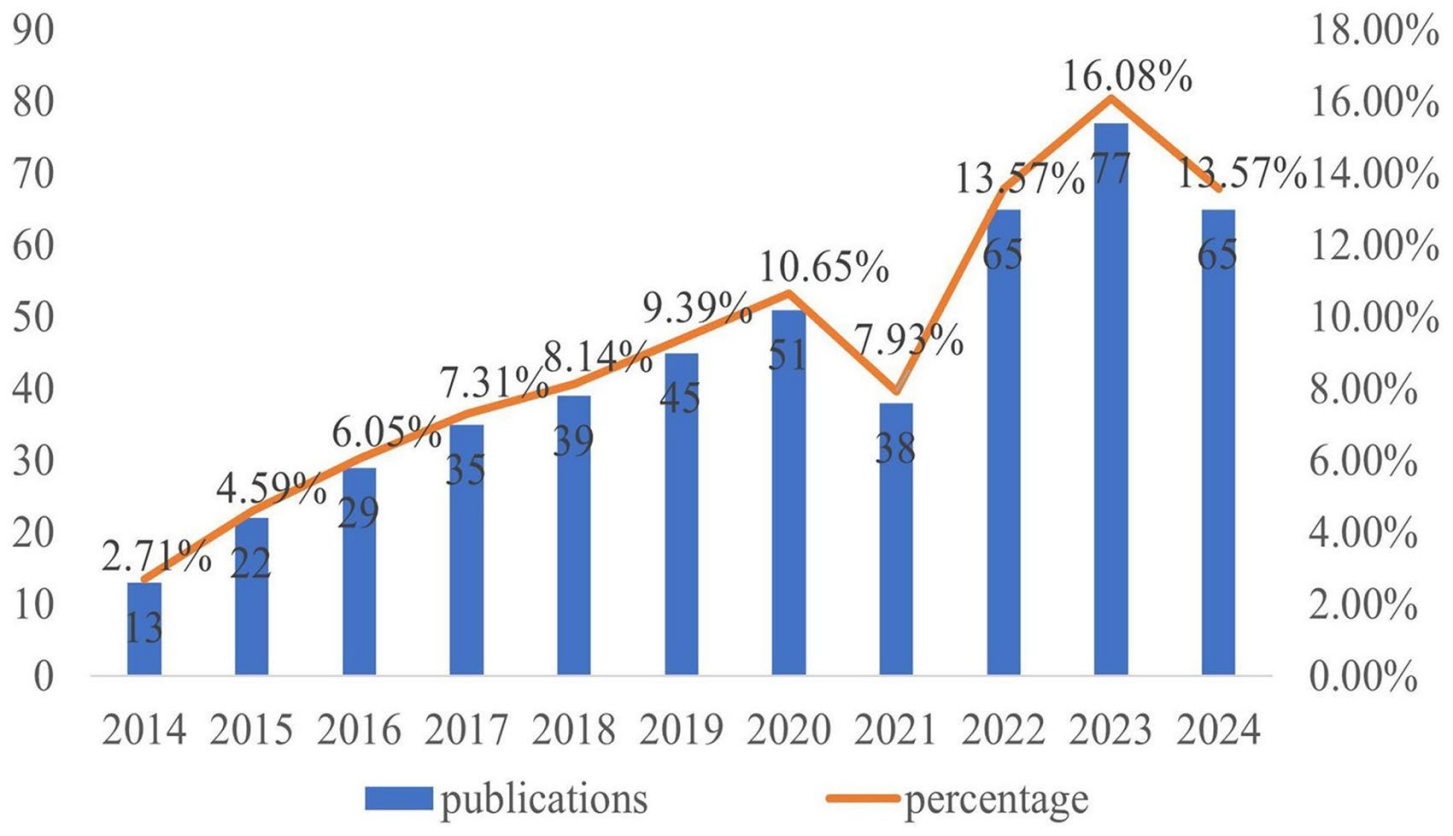
Document statistics on basic research of POCD from 2010 to 2024.

As illustrated in Figure 2, the number of papers focused on basic research of POCD has generally increased annually over the past decade, with a slight decline in 2021, and a peak in 2023. Notably, the papers published in the last 3 years account for approximately 43% of the total publications from the past decade, indicating that basic research on POCD has emerged as a hotspot and garnered increasing attention from scholars. However, since this study only included literature published before October 30, 2024, it is anticipated that the number of papers published in 2024 will continue to rise.

### 3.2 Author, journal, country, and institution information

#### 3.2.1 Author information

Author analysis can identify the prominent scholars and core research contributors within a specific field. The renowned scholar Derek J. de Solla Price observed that within a given research topic, a significant portion of papers are authored by a relatively small group of highly productive researchers. He noted that the size of this group is approximately equal to the square root of the total number of authors involved. This principle has since been referred to as Price’s law (de Solla Price et al., 1986), that is


(1) $$\overset{I}{\underset{m+1}{\sum }}n\left(x\right)=\sqrt{n}$$

In Equation 1, (*n*(*x*)) represents the number of authors who have published (*x*) papers, whereas *i* = *n*_max_ denotes the number of papers authored by the most prolific researchers in the field (with *n*_max_ = 27 based on VOSviewer analysis). Here, (*n*) represents the total number of authors, and (*M*) is the minimum number of papers published by core authors. According to Price’s law, the minimum number of papers required for an author to be considered among the core contributors is as follows:


(2) $$m\approx 0.749\times \sqrt{(N_{\text{max}}})$$

**Figure 3:**
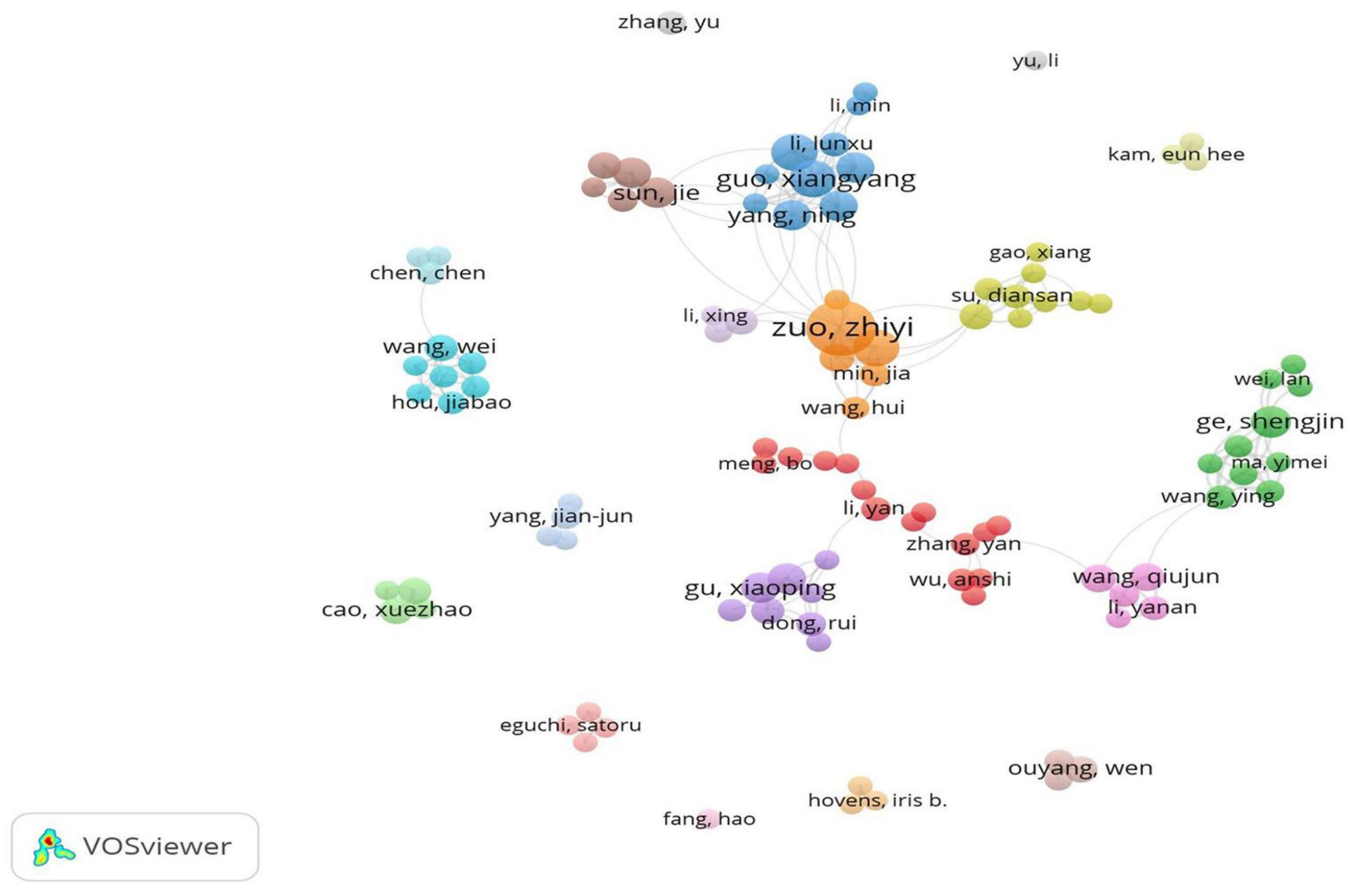
Visualization analysis of all core authors.

According to Equation 2, *m* ≈ 3.89. Thus, authors with four or more published articles (including those with exactly four) are classified as core authors in this field, resulting in a total of 105 core authors. Figure 3 visualizes these core authors via VOSviewer. In the figure, larger circle nodes correspond to authors with a greater number of publications. The connections between nodes indicate the strength of collaboration, with thicker lines representing a greater number of co-authored papers between two authors. Additionally, the color of the nodes signifies different clusters. It is evident that several collaborative groups among authors have emerged; however, intergroup collaboration remains relatively limited.

Table 1 presents the top 10 high-yield authors in the field of basic research on POCD, covering the period from January 1, 2014, to October 30, 2024. Among these highly productive authors, Professor Zuozhiyi, an anesthesiology professor at the University of Virginia in the United States, holds the top position, with a total of 27 published papers and 820 citations, with an average of 30.37 citations per paper. Professor Zuozhiyi is dedicated to research on brain protection and POCD and has achieved significant international recognition for his contributions. Professors Guoxiangyang and Lizhengqian, both affiliated with the Department of Anesthesiology at the Third Hospital of Peking University, rank second and third, respectively. They have frequently collaborated on research focused on perioperative brain health and brain function. These studies investigated the potential relationships between POCD and related signaling molecules in the hippocampus, such as hypoxia-inducible factor-1 alpha (HIF-1α) and vascular endothelial growth factor (VEGF) (Cao et al., 2019; Cao et al., 2018), as well as the effects of isoflurane anesthesia on POCD in elderly rats (Ni et al., 2015). Notably, most of the top 10 authors are Chinese researchers, highlighting their emphasis on elucidating the molecular mechanisms underlying POCD.

**Table 1:** Top 10 contributing authors in POCD basic research.

Rank	Author	Documents	Citations	Average citation/publication
1	Zuozhiyi	27	820	30.37
2	GuoXiangyang	13	345	26.54
3	LiZhengqian	13	345	26.54
4	Li jun	13	316	24.31
5	GeShengjin	10	100	10
6	CaoYiyun	9	256	28.44
7	Yangning	9	226	25.11
8	Nicheng	9	290	32.22
9	Sunjie	9	390	43.33
10	GuXiaoping	9	170	18.89

#### 3.2.2 Journal information

Basic research on POCD over the past decade has been published primarily in medical journals, with a few exceptions for comprehensive journals. Notably, journals focused on neuromedicine and geriatrics are predominant. Table 2 lists the top 10 journals in this field, all of which are medical journals. Among them, the “Journal of Neuroinflammation” stands out as the leading publication, with a total of 22 articles and the highest impact factor among the top 10 journals. In addition to the “Molecular Medicine Reports” (medicine, research, and experimental), “Experimental and Therapeutic Medicine” (medicine, research, and experimental), and “Aging US” (cell biology and genetics), the remaining seven journals also encompass various aspects of neuroscience. Citation analysis revealed that the “Journal of Neuroinflammation” not only has the highest number of published articles but also has the highest total number of citations, suggesting that high-quality of research is disseminated in this journal and that significant attention within the field is given to basic research on POCD. The “Behavioral Brain Research” journal ranks second in terms of citations and citations per article, also indicating that this topic has garnered considerable interest from the research community.

**Table 2:** Top 10 journals with the highest publication output in POCD basic research.

Rank	Source	Publications	Citations	Average citation/publication	JCR	Impact factor
1	Journal of Neuroinflammation	22	1,072	48.73	Q1	9.3
2	Behavioral Brain Research	14	359	25.64	Q2	2.6
3	Molecular Medicine Reports	14	245	17.5	Q2	3.4
4	Molecular Neurobiology	13	203	15.62	Q1	4.6
5	Frontiers in Aging Neuroscience	12	211	17.58	Q2	4.1
6	Experimental and Therapeutic Medicine	12	132	11	Q3	2.4
7	Brain Research Bulletin	11	110	10	Q2	3.5
8	Neurochemical Research	11	99	9	Q2	3.7
9	CNS Neuroscience & Therapeutics	11	165	15	Q1	4.8
10	Aging-US	10	208	20.8	Q2	3.9

**Figure 4:**
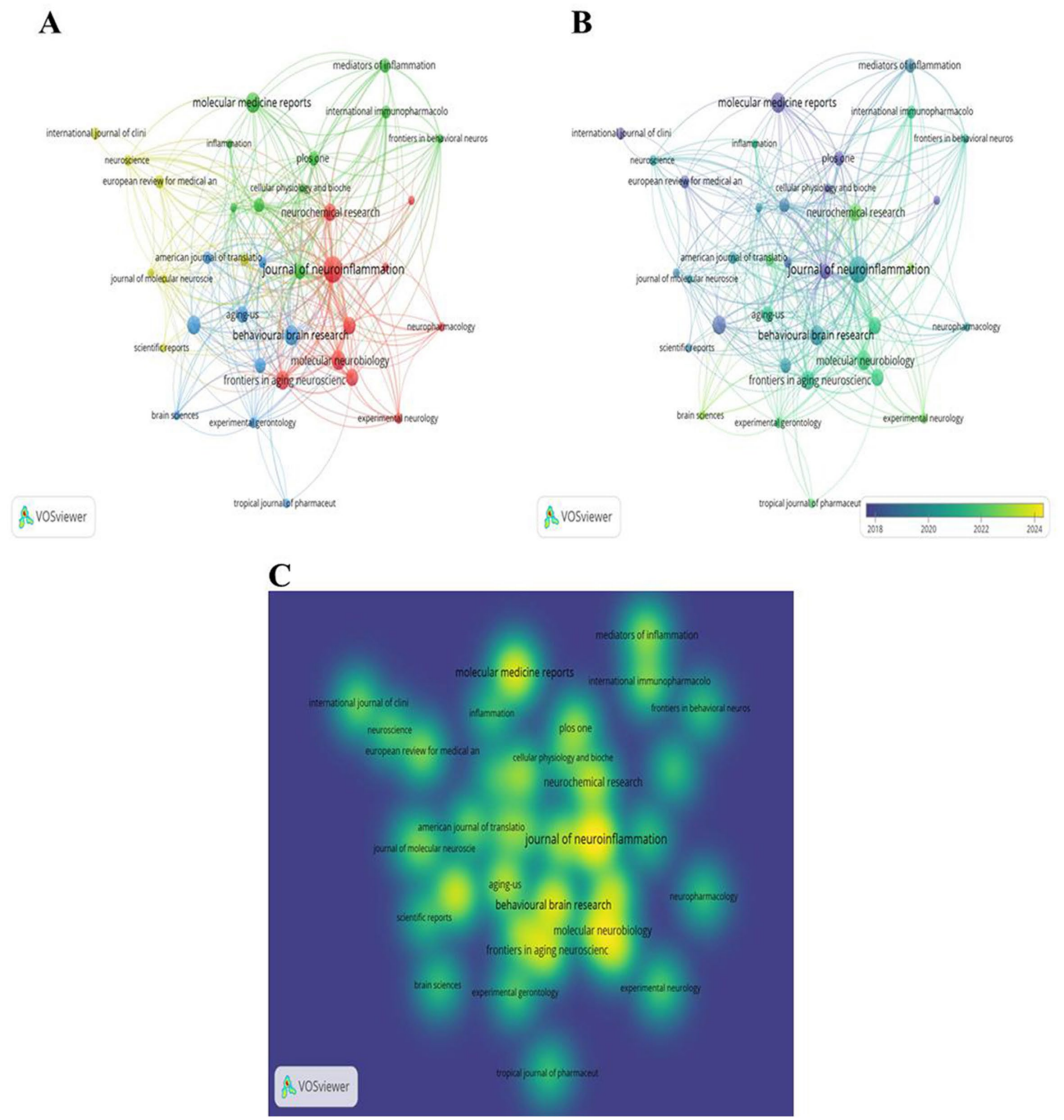
**(A)** Co-occurrence network view of journals **(B)** time view; **(C)** density view.

Using VOSviewer, we visually analyzed journals with at least four publications, identifying a total of 36 journals that met the criteria, as illustrated in Figure 4. (A) The graph presents the co-occurrence network view of these journals, with different colors representing distinct clusters. The red clusters are predominantly composed of journals within the field of neuroscience, whereas the green clusters mainly include journals focusing on immunology and cell biology. The blue and yellow clusters encompass a variety of disciplines, including neuroscience, geriatrics, behavioral medicine, pharmacology, and anesthesiology. (B) The timeline chart shows the publication activity of 34 journals, revealing that since approximately 2020, neuroscience journals have increasingly become the preferred choice for scholars in this domain. (C) The density view of the 34 journals is depicted in the figure, where the brightness of the colors reflects the number of published papers. A brighter color indicates a greater volume of publications. Notably, the three journals with the most publications are the “Journal of Neuroinflammation,” “Behavioral Brain Research,” and “Molecular Medicine Reports.”

#### 3.2.3 Country and institutional information

To identify which countries have made the most significant contributions to basic research on POCD, this study analyzed the number of papers published by 13 countries. Table 3 presents the top five countries in terms of number of publications. Chinese scholars have led the field, with an impressive total of 444 articles published over the past decade, significantly outpacing other countries. This highlights the extensive attention given to POCD research by Chinese scholars during this period. The United States ranks second, with a total of 56 articles published. Notably, among the top 5 countries, only China and the United States have published more than 10 articles, indicating a stark imbalance in the distribution of research contributions within this field, with a few countries dominating the output. In terms of citations, the published works of Chinese scholars have garnered a remarkable total of 7,713 citations; however, the average citations per article stand at only 17.37, placing China fourth among the top five countries in this regard. In contrast, Dutch scholars, despite publishing only five articles, achieved an average of 71.8 citations per article, ranking first for this metric. This result suggests that the literature produced by Dutch scholars is recognized for its authority and impact within the research community.

**Table 3:** Top five countries by publication output in POCD basic research.

Rank	Country	Documents	Citations	Average citation/publication
1	China	444	7,713	17.37
2	United States	56	1,615	28.84
3	Japan	9	188	20.89
4	South Korea	8	112	14
5	The Netherlands	5	359	71.8

**Figure 5:**
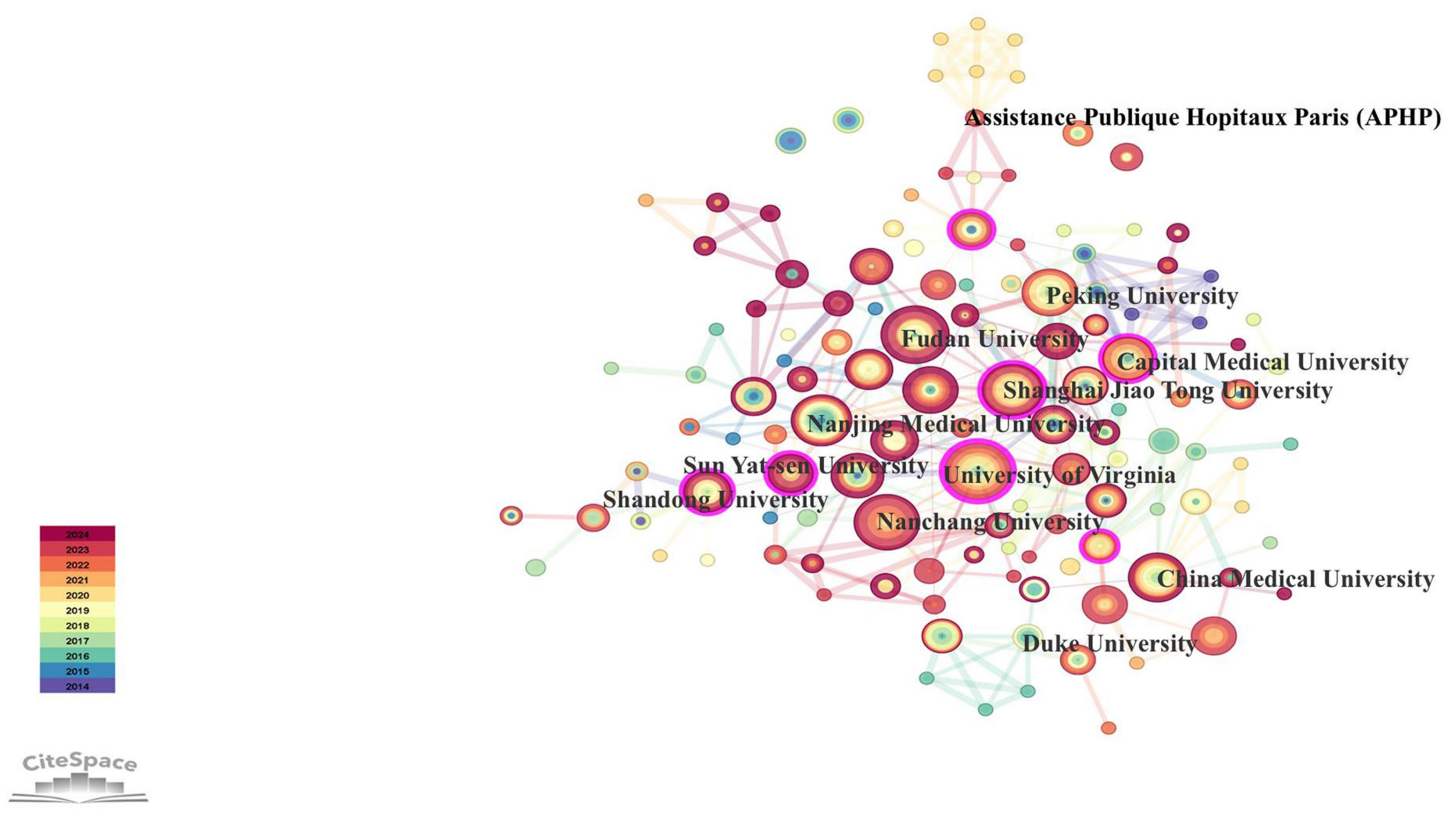
Cooperation network view of document publishing institutions.

Analysis of the affiliations of authors in this field was performed, and Table 4 presents the top 10 organizations on the basis of publication output. Each of these institutions has published more than 15 articles, with the University of Virginia in the United States ranking first. Notably, nine of the top 10 institutions are located in China, highlighting the significant interest in this research area among Chinese scholars. Additionally, we utilized CiteSpace to analyze the collaboration networks of these publishing institutions, with the results displayed in Figure 5. In the figure, larger nodes represent a greater number of published papers, and the gradual color change of the nodes indicates the year of publication, spanning from 2014–2024, as reflected in the legend in the lower left corner. The University of Virginia is at the center of the largest institutional collaboration network, extending its connections to Fudan University, Peking University, Shanghai Jiao Tong University, and other Chinese institutions. Furthermore, China boasts the most extensive collaboration network, with Peking University, Shanghai Jiao Tong University, Fudan University, Nanchang University, and Capital Medical University each maintaining their collaborative links. Additionally, Duke University and Assistance Publique-Hôpitaux de Paris (APHP) also feature distinct collaboration networks.

**Table 4:** Top 10 institutions by publication output in POCD basic research.

Rank	Organization	Documents	Citations	Average citation/publication
1	University of Virginia	26	818	31.46
2	Fudan University	25	329	13.16
3	Nanchang University	24	354	14.75
4	Nanjing Medical University	24	873	36.38
5	Shanghai Jiao Tong University	23	408	17.74
6	China Medical University	20	325	16.25
7	Sun Yat-sen University	17	537	31.59
8	Peking University	17	452	26.59
9	Capital Medical University	16	307	19.19
10	Shandong University	15	350	23.33

### 3.3 Keyword information analysis

**Figure 6:**
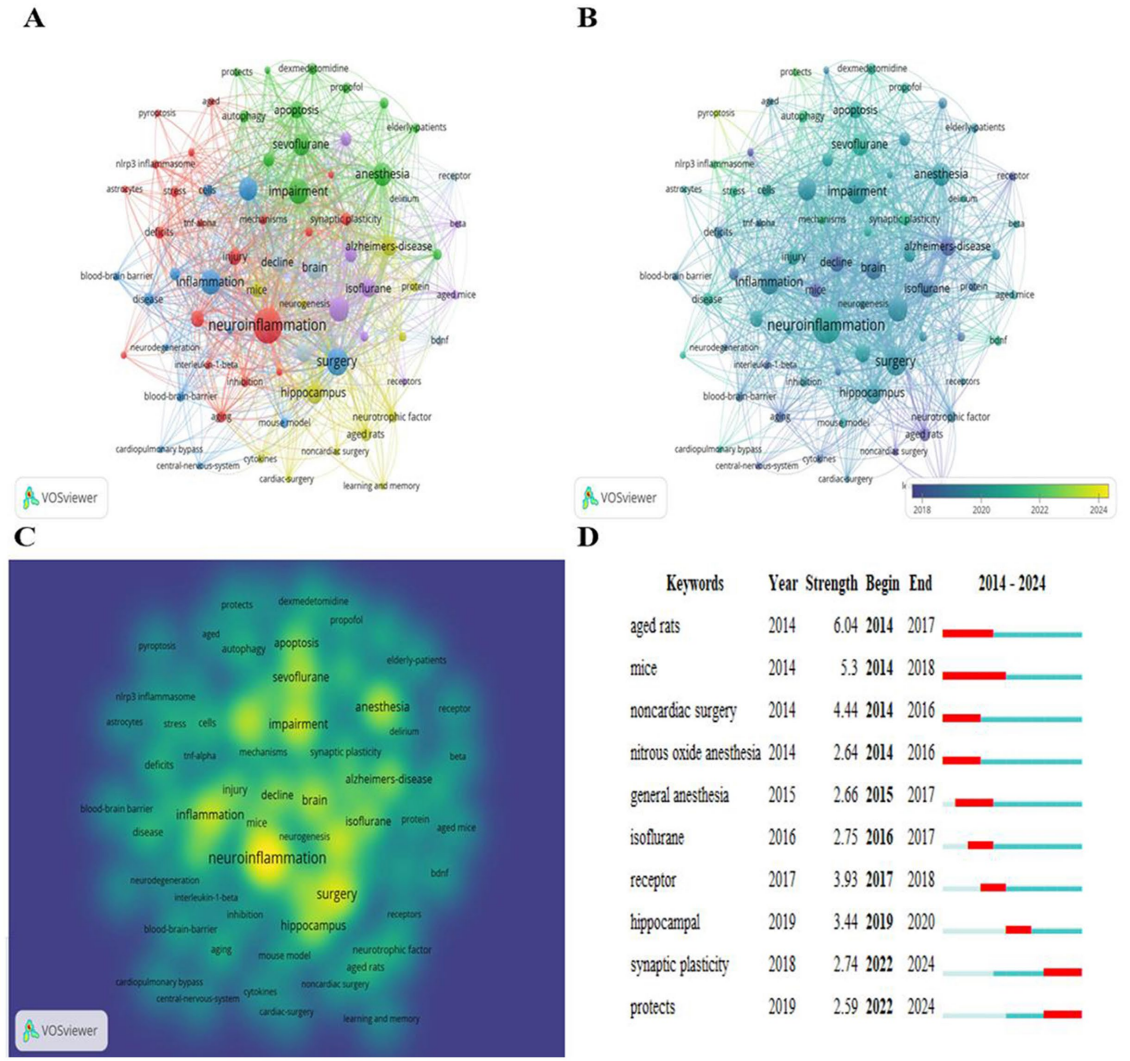
**(A)** Co-occurrence network view of keywords, **(B)** time view; **(C)** density view; **(D)** top 10 keywords with explosive intensity.

Keywords encapsulate the essence and themes of an article. The co-occurrence analysis of keywords can reveal hotspots within a research field. In this study VOSviewer was employed to analyze keywords from 479 published works with a focus on those that appeared 10 times or more. Since “postoperative cognitive dysfunction” as a keyword is inevitably the most frequently used keyword, “postoperative cognitive dysfunction” and some high-frequency synonyms were deleted in this analysis to avoid their impact on the statistical results. A total of 72 keywords were selected and the analysis results are shown in Figure 6A. The circular node represents keyword frequency. The larger the node is the more the keyword appears and the more it can represent hot spots in the field. The node connection represents the correlation strength. The thicker the connection is the more frequently keywords appear concurrently in a document. The node color represents different clusters that is the research topic. The red and blue clusters focus mainly on the risk factors and potential mechanisms of POCD, respectively, such as the role of some signaling molecules in POCD. The green cluster involves mainly the inducement of POCD and the research linking general anesthesia drugs (such as dexmedetomidine propofol and sevoflurane) and POCD. The yellow cluster focuses mainly on the research linking POCD and changes in the nervous system. The purple and gray clusters primarily investigate the common factors in basic research on POCD. Figure 6B shows the time distribution view of the basic research field on POCD. In the past decade basic research on POCD has focused on incentives and risk factors and has gradually transitioned to etiological research such as enhanced hippocampal neuroinflammation and synaptic plasticity damage and then to specific molecular mechanism research. The current research focuses on cell death and POCD. Figure 6C shows the density view. The brighter the color is the more frequently the keywords appear. The five most frequent keywords are “neuroinflammation,” “surgery,” “impairment,” “memory,” and “inflammation.”

The term explosive words refers to the phenomenon of a keyword frequently appearing in a specific period. This phenomenon can not only reflect the evolution of research hotspots over time but also the development of research trends in recent years and may indicate future research directions. Therefore, using the explosive word detection function of CiteSpace, we calculated the top 10 keywords with explosive intensity, which are shown in Figure 6D. Research has shown that elderly patients are at high-risk group for POCD, and old age is also the most common high-risk factor for POCD (Rundshagen, 2014). Elderly rats are often used as ideal models to study cognitive decline in elderly patients because of their similar performance to that of elderly humans in term of cognitive function, learning and memory ability, and physiological changes. Therefore, “aged rats” has become one of the keywords with the highest outbreak intensity. Between 2014 and 2016, “noncardiac surgery” and “nitrous oxide anesthesia” became two keywords associated with increased outbreak intensity. Since 2015, research on the relationship between general anesthesia and POCD has become mainstream (Zhang J. et al., 2015; Zhang Y. et al., 2015), especially between inhalation general anesthesia and POCD (Wang et al., 2018). The establishment of animal models under isoflurane anesthesia has been favored by relevant scholars (Tanino et al., 2016; Li et al., 2017). In recent years, research on the deep molecular mechanism of POCD has gradually increased, and research on the role of hippocampal receptor proteins and signaling pathways in POCD has also significantly increased (Li et al., 2022; Qiu et al., 2020). Moreover, “synaptic plasticity,” an important mechanism of POCD, has been explored by many scholars. Studies have shown that SIRT3 may alleviate POCD in elderly mice by alleviating synaptic plasticity damage and inhibiting hippocampal neuritis (Liu et al., 2021). In addition, a study by Wu et al. (2024) revealed that a decrease in SIRT1/BDNF levels in the hippocampal CA1 region led to the impairment of synaptic plasticity in elderly mice, which may be an important factor leading to POCD.

### 3.4 Analysis of co-citation

VOSviewer was used to analyze the co-cited journals in this research field. Table 5 shows the journals with the top 5 rates of co-citation. Each of the five journals had a co-citation count exceeding 400, and “Anesthesiology,” the top journal in the field of anesthesia, ranked first.

**Table 5:** Top five journals most frequently co-cited in POCD basic research.

Rank	Source	Citations	JCR	Impact factor
1	Anesthesiology	915	Q1	9.1
2	Journal of Neuroinflammation	522	Q1	9.3
3	Brain Behavior and Immunity	503	Q1	8.8
4	Journal of Neuroscience	459	Q1	4.4
5	PLoS One	442	Q1	2.9

**Figure 7:**
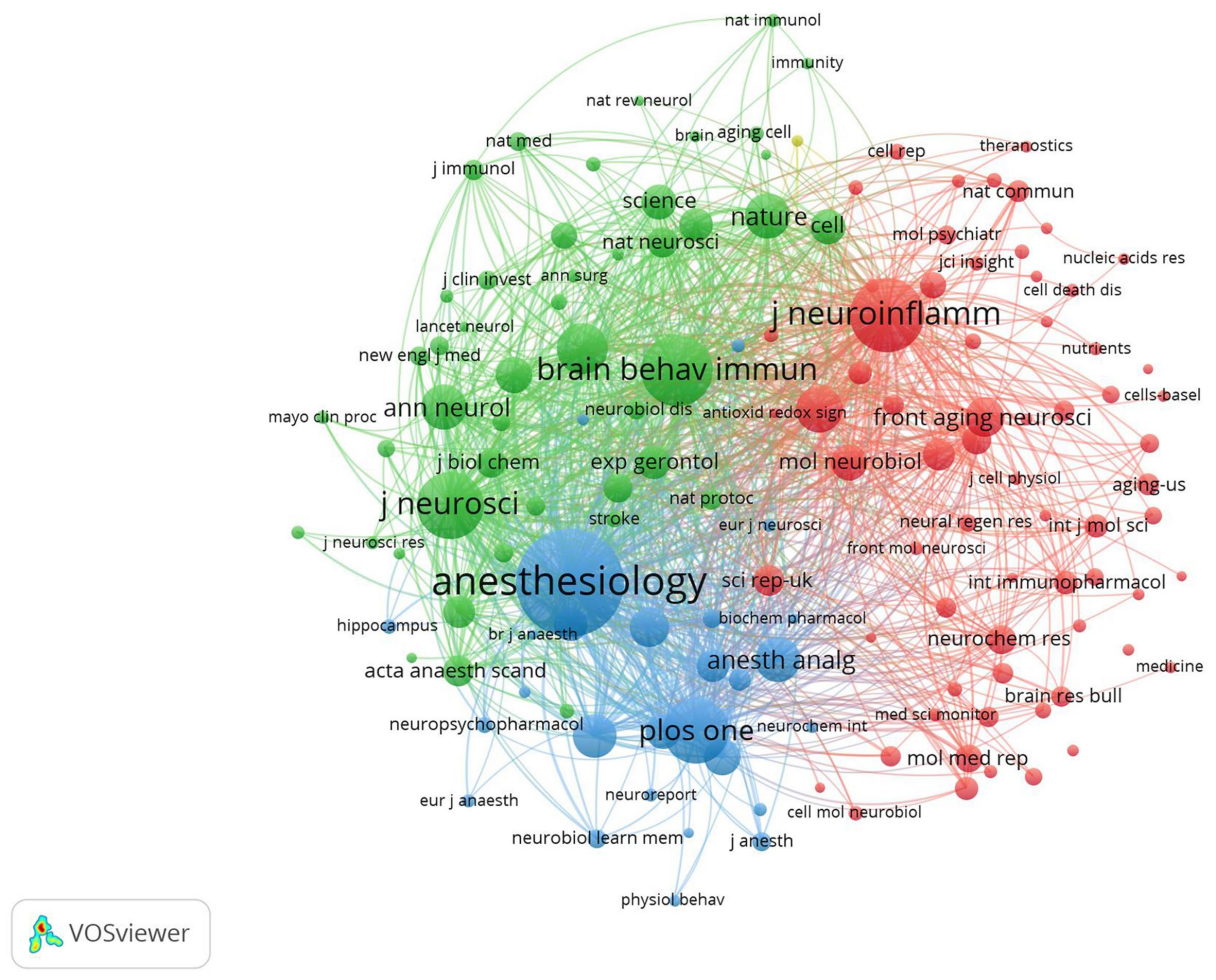
Clustering network view of co-cited journals.

Figure 7 is a cluster view of 141 co-cited journals with 30 or more citations. Different color regions in the figure represent different clusters. The figure shows that the cited journals can be divided into four approximate clusters, with the red cluster being the largest. The journals cover a wide range of disciplines, including cell biology, molecular biology, neuroscience, and pharmacology. The main purpose of citing these journals is to provide theoretical support for research in multiple fields and disciplines. Green clusters comprise the second-largest cluster. Most of the journals included are high-quality and authoritative journals, including “Cell,” “Nature,” and “Science,” which are recognized as the three major journals in the global academic field, and the “New England Journal of Medicine” and “Lancet,” which are well known in the medical field. The purpose of quoting these journals is to reflect the most authoritative and advanced research directions. Blue and yellow clusters contain fewer journals. The blue cluster refers mainly to certain journals in the fields of anesthesia and neuromedicine, whereas the yellow cluster includes only includes “Journal of the American Medical Directors Association.”

**Figure 8:**
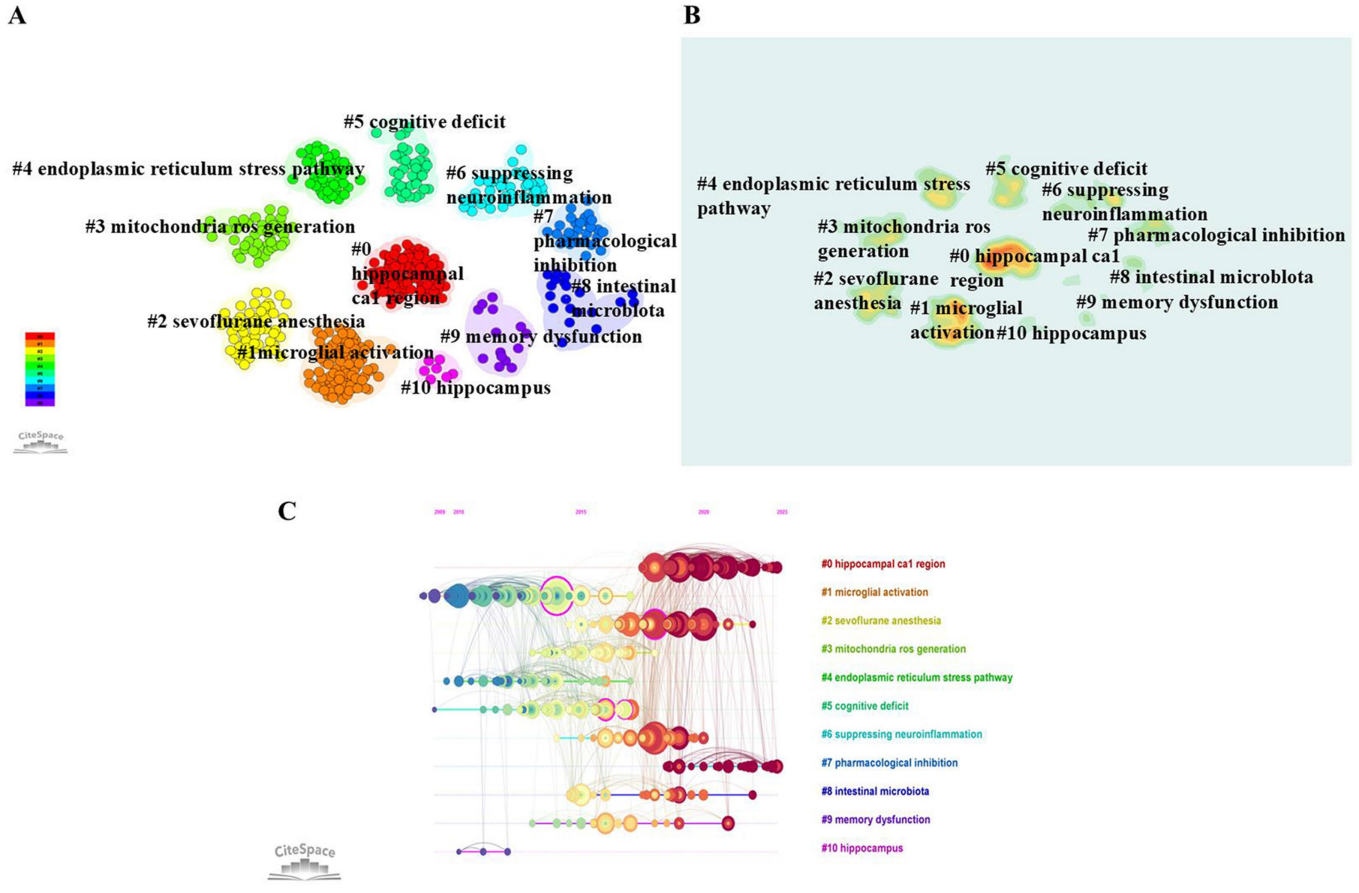
**(A)** Clustering information of co-cited literature; **(B)** clustering heat map; **(C)** timeline view of co-cited literature.

**Figure 9:**
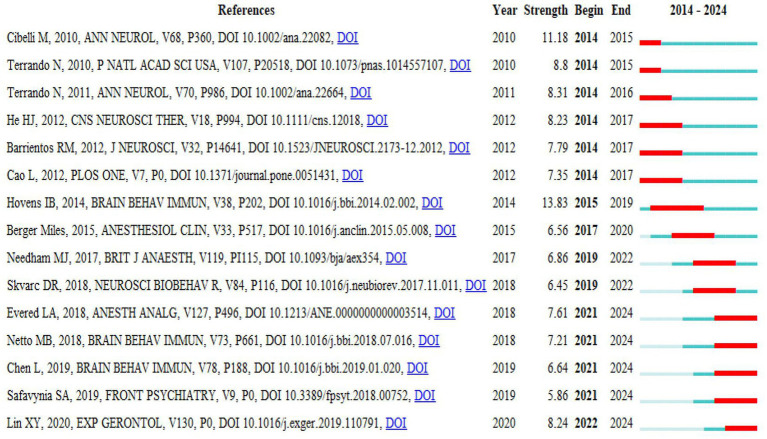
Co-cited references with burst intensity in the top 15.

Figure 8 shows the clustering analysis of all co-cited literature by CiteSpace, where (A) is the clustering view of co-cited literature, (B) is the corresponding heatmap, and (C) is the timeline view of co-cited literature, in which the co-cited literature is divided into 11 clusters. “Microglial activation,” “mitochondria ROS generation,” the “endoplasmic reticulum stress pathway” and “suppressing neuroinflammation” are all related to the mechanisms of POCD. In Figure 8C, each circular node in the figure represents a reference, and the position of the node on the horizontal axis corresponds to the time at which the reference was first cited. The larger the node is, the more times the reference was co-cited. The node color represents the time at which the reference was cited. It corresponds to the legend in the lower left corner, while the cluster label on the right represents the subject of the field. “Microglial activation,” the “endoplasmic reticulum stress pathway,” and “cognitive deficit” were the first research hotspots, and “hippocampal CA1 region” and “pharmaceutical innovation” are the current research hotspots. In addition, according to Figure 8B, the “hippocampal CA1 region” is the cluster with the most references. The explosive analysis of cited references can reflect the importance of the literature and the time at which it is most popular. Figure 9 shows the top 15 references in terms of burst intensity. The popularly cited literature on basic research on POCD in the last 3 years is related mainly to research on the specific mechanisms of POCD, especially the role of neuroinflammation in POCD.

Table 6 shows the top 10 co-cited references, and analyzes the sources, impact factors, research types, and research results of the references.

**Table 6:** Top 10 most frequently co-cited references in POCD basic research.

Rank	Title	Citations	Source	IF	Research type	Research conclusion
1	Long-term postoperative cognitive dysfunction in the elderly ISPOCD1 study. ISPOCD investigators. International Study of Post-Operative Cognitive Dysfunction	126	Lancet	98.4	Multicenter study	The risk factors for early postoperative cognitive dysfunction were increased age and duration of anesthesia, low education level, second operation, postoperative infection, and respiratory complications. Age was the risk factor for late postoperative cognitive dysfunction. Hypoxemia and hypotension were not important risk factors (Moller et al., 1998)
2	Long-term consequences of postoperative cognitive dysfunction	125	Anesthesiology	9.1	Multicenter comparative study	Cognitive dysfunction after noncardiac surgery is associated with increased mortality, the risk of leaving the labor market prematurely, and dependence on social transfer payments (Steinmetz et al., 2009)
3	Role of interleukin-1β in postoperative cognitive dysfunction	119	Annals of Neurology	8.1	Basic research	A peripheral surgery-induced innate immune response triggers an IL-1β-mediated inflammatory process in the hippocampus that underlies memory impairment. This may represent a viable target to interrupt the pathogenesis of postoperative cognitive dysfunction (Cibelli et al., 2010)
4	Predictors of cognitive dysfunction after major noncardiac surgery	90	Anesthesiology	9.1	A prospective longitudinal study	Cognitive impairment is common in adult patients of all ages at discharge after major non-cardiac surgery, but only the elderly (60 years old or above) are at significant risk of long-term cognitive problems. Patients with POCD have an increased risk of death in the first year after surgery (Monk et al., 2008)
5	Tumor necrosis factor-alpha triggers a cytokine cascade yielding postoperative cognitive decline	89	Proceedings of the National Academy of Sciences of the United States of America	9.4	Basic research	Peripheral blockade of TNF-α can limit the release of IL-1 and prevent neuroinflammation and cognitive decline in POCD mice. In addition, early treatment with anti-TNF antibody can prevent cognitive decline caused by surgery (Terrando et al., 2010)
6	Postoperative cognitive dysfunction: involvement of neuroinflammation and neuronal functioning	76	Brain Behavior and Immunity	8.8	Basic research	The changes in postoperative neuroinflammation, BDNF, and neurogenesis may be the potential mechanism of POCD (Hovens et al., 2014)
7	Resolving postoperative neuroinflammation and cognitive decline	71	Annals of Neurology	8.1	Basic research	Peripheral surgery destroys the blood-brain barrier by activating tumor necrosis factor-α (TNF α)/nuclear factor (NF)-κB, which makes peripheral macrophages migrate to the hippocampus, and then promotes the neuroinflammatory reaction of memory impairment. The activation of the nicotinic acetylcholine receptor α 7 subtype is an endogenous inflammatory relief pathway, which can prevent TNF-α-induced NF-κB activation, macrophage migration to the hippocampus, and postoperative cognitive decline (Terrando et al., 2011)
8	Neuroinflammation and cognitive function in aged mice following minor surgery	52	Experimental Gerontology	3.3	Basic research	The increase of proinflammatory cytokines in the hippocampus of elderly mice after surgery may lead to common cognitive deficits in elderly mice after surgery, and reducing the neuroinflammation caused by surgery may help to prevent the decline of cognitive ability in elderly patients after surgery (Rosczyk et al., 2008)
9	Morris water maze: procedures for assessing spatial and related forms of learning and memory	51	Nature Protocols	13.1	Basic research	Morris water maze can be used to evaluate spatial-related programs and various forms of learning and memory (Vorhees and Williams, 2006)
10	Postoperative impairment of cognitive function in rats: a possible role for cytokine-mediated inflammation in the hippocampus	50	Anesthesiology	9.1	Basic research	The transient decline of neurocognitive ability caused by surgery is temporarily related to the activation of glial cells and the increase of pro-inflammatory cytokines in the hippocampus. Alleviating central nervous inflammation may prevent the occurrence of postoperative cognitive dysfunction (Wan et al., 2007)

## 4 Discussion

In recent years, with the progress of anesthesia/surgery concepts and technology, POCD has received increasing attention worldwide. At the same time, there is increasing research on POCD. POCD is a complication after anesthesia/surgery characterized by memory impairment, decreased information processing ability, and inattention (Lin et al., 2020). Currently, the identified risk factors for POCD include advanced age, low educational level, alcohol abuse, depression, type and duration of surgery, and anesthesia methods (Bhushan et al., 2021). The purpose of this study is to investigate the relevant literature on basic research on POCD published in the past decade; review the development trends of this research field; discuss and analyze the core authors in this research field, high- productivity countries, and institutions, key journals, high-frequency keywords, and co-citations; and provide a visual map. Through the bibliometric analysis of basic research on POCD, this study can provide researchers with a general understanding of POCD.

According to the analysis of the authors, journals, countries, and institutions in this research field, there are 105 core authors in total, and relatively stable author collaboration groups have formed. Professor Zuozhiyi, a professor in the Department of Anesthesiology at the University of Virginia, is the most prolific author. Among the top 10 journals with the largest number of articles, most belong to the field of neuroscience. “The Journal of Neuroscience” has the most papers published in this field, and its number of overall citations and number of citations per article are the highest. Chinese scholars have contributed the most papers, but their number of citations per article is very low, which demonstrates that Chinese scholars still need to improve the authority and recognition of the published literature. In terms of the number of citations per article, the literature published by Dutch scholars has the highest recognition and authority. Among the top 10 institutions with most publications, except for the University of Virginia, the other nine institutions are located in China. There have been many instances of collaboration between the issuing agencies in this research field, and a stable collaboration network has formed. China has the most extensive institutional collaboration network.

The analysis of high-frequency keywords identified, several stable research topics in this field; “neuroinflammation,” “surgery,” “impairment,” “memory,” and “inflammation” are the five keywords with the highest frequency. In recent years, many studies have shown that the enhancement of neuroinflammatory response may be an important potential mechanism of POCD, including the involvement of a variety of receptors and related signaling molecules. A study by Sun et al. (2022) and other scholars in 2021 revealed that electroacupuncture may alleviate the related neuroinflammatory response by inhibiting NLRP3 inflammatory bodies, thus improving POCD in elderly mice. Another recent study published in the “Journal of Neuroinflammation” reported that 4-octyl itaconate can limit neuroinflammation and promote neurogenesis by stabilizing the intestinal microbiota and activating Nrf2 signal transduction, thereby reducing POCD (Kong et al., 2024). In addition, since 2024, research on the relationship between pyroptosis and POCD has gradually increased and has become a hot spot in basic research on POCD. Shen et al. (2024) reported that the intraperitoneal injection of carnosine can inhibit NLRP3 mediated astrocyte apoptosis and neuroinflammation to improve POCD in elderly rats. In contrast, hippocampal histone deacetylase 6 (HDAC6) can induce microglial cell death by activating NLRP3 inflammatory bodies, hence contributing to POCD in elderly mice. In short, whether it is neuroinflammation or cell death, research on the specific molecular mechanisms of POCD has remained the focus of current research and is likely to continue, even in the future.

On the basis of the co-citation statistics for the basic research literature on POCD from the past decade, “Anesthesiology” is the most frequently cited journal; “Long-term postoperative cognitive dysfunction in the elderly ISPOCD1 study. ISPOCD investigators. International Study of Post-Operative Cognitive Dysfunction” published in “Lancet” is the most frequently cited reference; and “Lancet” (IF: 98.4) is also the most influential journal among all the co-cited journals. The cluster of the “hippocampal CA1 region” contains the most co-cited studies, such as the study “Activation of astrocyte GQ pathway in hippocampal CA1 region attenuates anesthesia/surgery induced cognitive dysfunction in aged mice” published by Wang et al. (2022) in “Frontiers in Aging Neuroscience,” which reveals that the activation of the GQ pathway in hippocampal CA1 region astrocytes can improve the learning and memory ability of POCD aged mice and synaptic plasticity. Notably, recent advances in basic research are increasingly driving clinical translation. For example, clinical studies have identified neuroinflammation-related biomarkers, such as CRP and IL-6, as potential indicators of POCD (Zhang J. et al., 2015; Zhang Y. et al., 2015). Additionally, elevated preoperative serum levels of phosphorylated neurofilament-H have been validated as a biomarker for POCD in elderly patients undergoing hip replacement surgery (Zhang et al., 2019). These findings offer novel insights for the clinical diagnosis and early intervention of POCD. Nevertheless, the sensitivity and specificity of current biomarkers remain suboptimal, underscoring the need for future studies to integrate multiomics approaches for the identification of reliable diagnostic markers for POCD.

From a bibliometric perspective identifying the relevant research context for basic research on POCD is highly valuable. For example, the analysis of high-frequency keywords in this paper can provide scholars with the focuses and hot spots in this research field and provide ideas for their research topics. The analysis of core authors and journals with a large number of publications provided in this paper can help scholars quickly find the literature they want to reference in research and provide some suggestions for scholars on selecting journals when creating papers on this topic.

In addition, this study has several limitations. First, this study selected only the Web of Science Core Collection (WOSCC) as the literature data source, and the citation index was Science Citation Index Expanded, excluding other databases (such as Scopus), which inevitably leads to the problem of incomplete data. Second, when analyzing and interpreting the data, researchers need to have a comprehensive and in-depth understanding of the field; otherwise, subjectivity inevitably arises. Research on basic research on POCD still has significance and value for further exploration. Therefore, in future research, it will be necessary to integrate the literature from multiple databases to make the selected literature more comprehensive. Furthermore, it is necessary to actively communicate with relevant scholars in the field, understand the frontier dynamics of the field, improve the objective cognition regarding the field, and form an objective and rational cognition concerning the field.

## Data Availability

The original contributions presented in the study are included in the article/supplementary material, further inquiries can be directed to the corresponding authors.
